# Atractylenolide-I Sensitizes Human Ovarian Cancer Cells to Paclitaxel by Blocking Activation of TLR4/MyD88-dependent Pathway

**DOI:** 10.1038/srep03840

**Published:** 2014-01-23

**Authors:** Jian-Ming Huang, Guo-Nan Zhang, Yu Shi, Xiao Zha, Yi Zhu, Miao-Miao Wang, Qing Lin, Wen Wang, Hai-Yan Lu, Shi-Qi Ma, Jia Cheng, Bi-Fang Deng

**Affiliations:** 1 Department of Gynaecologic Oncology, Sichuan Cancer Hospital, No. 55, Section 4, South People's Road, Chengdu 610041, Sichuan, P. R. China; 2 Department of Biochemistry & Molecular Biology, Sichuan Cancer Institute, Chengdu 610041, Sichuan, P. R. China; 3 Graduate School, Guangxi Medical University, Nanning 530021, Guangxi, P. R. China; 4 Department of Ultrasound, Sichuan Cancer Hospital, Chengdu 610041, Sichuan, P. R. China

## Abstract

Paclitaxel, a known TLR4 ligand, leads to activation of TLR4/MyD88-dependent pathway that mediates chemoresistance and tumor progression in epithelial ovarian carcinoma (EOC). Atractylenolide-I (AO-I), a novel TLR4-antagonizing agent, inhibits TLR4 signaling by interfering with the binding of LPS or paclitaxel to membrane TLR4 of human leukocytes. In this study, AO-I was found to attenuate paclitaxel-induced protein expression of IL-6, VEGF and survivin, and to enhance early apoptosis and growth inhibition in MyD88^+^ EOC cells; AO-I was shown to fit into the hydrophobic pocket of human MD-2 and to partially overlap with the binding site of paclitaxel by docking simulations, suggesting that AO-I may block the MD-2-mediated TLR4/MyD88-dependent paclitaxel signaling in MyD88^+^ EOC cells. Therefore, AO-I could significantly sensitize the response of MyD88^+^ EOC cells to paclitaxel by blocking MD-2-mediated TLR4/MyD88 signaling, and that AO-I-paclitaxel combination could be a promising strategy for the treatment of EOC with a functional TLR4/MyD88/NF-κB pathway.

Epithelial ovarian cancer (EOC) is the leading cause of death among gynecological malignancies worldwide and usually has a poor prognosis^1^,^2^. Recent studies revealed that EOC cells expressing TLR4 and MyD88 constitutively secrete pro-inflammatory cytokines and are resistant to the paclitaxel, and directly contributes to their own survival and tumor progression^3^,^4^,^5^,^6^. Our previous study reported that MyD88 expression was observed in 77.1% of patients with EOC, which is an independent prognostic factor for poor disease-free survival and overall survival for EOC^4^.

TLR4, the receptor for lipopolysaccharide, is unique in that it activates both MyD88-dependent and TRIF-dependent or MyD88-independent pathways. MyD88 is an adaptor protein for TLR4 signaling known to hyper-activate NF-κB, MAPK and PI3K pathways driving tumor survival and paclitaxel chemoresistance in EOC cells^7^,^8^,^9^,^10^,^11^. Paclitaxel is an important chemotherapeutic agent against EOC which acts by microtubule over-stabilization. However, paclitaxel elicits both cytotoxic and pro-survival responses in tumor cells. The likely mechanism for paclitaxel-dependent tumor-activating effects is the ability of paclitaxel to activate TLR4/MyD88 signaling pathway. Paclitaxel, a known TLR-4 ligand, enhances NF-κB activity and up-regulates expression of X-linked inhibitor of apoptosis (XIAP) and pro-inflammatory cytokines known to promote tumor survival and progression in EOC. The expression of pro-inflammatory cytokines by MyD88^+^ EOC cells are lost upon the knockdown of MyD88, suggesting that an active MyD88-dependent TLR4 signaling is responsible for MyD88/NF-κB-mediated cytokine secretion, proliferation and paclitaxel resistance of EOC cells. TLR4/MyD88 signaling has become prominently implicated as a means by which EOC cells can acquire the ability to invade, disseminate and resist paclitaxel-induced apoptosis^3^,^12^,^13^.

The TLR4 accessory protein, myeloid differentiation protein 2 (MD-2), is known to be an essential component for the initiation of TLR4/MyD88 signaling. The binding of LPS or paclitaxel to human MD-2 is required for dimerization of human TLR4 leading to activation of the MyD88-dependent pathway. Formation of the TLR4/MD-2 complex by paclitaxel may suggest important new mechanisms for paclitaxel-resistant tumors^14^ and blocking the binding of paclitaxel to MD-2 may reduce its pro-survival response, then enhance its cytotoxic response. It is also important to identify alternative chemotherapy options that would benefit MyD88^+^ EOC patients.

Atractylenolide-I (AO-I) is a naturally occurring sesquiterpene lactone isolated from *Atractylodes macrocephala Koidz* [Family: Compositae], and has been used for anti-inflammatory purposes and the treatment of cancers^15^,^16^,^17^,^18^,^19^,^20^. Anti-inflammatory effect of AO-I displays a potent inhibitory effect on angiogenesis by a set of downregulation of NO, TNF-α, IL-1β, IL-6 and VEGF in monocytes and macrophages stimulated with LPS^21^,^22^. It has been reported that AO-I has a binding site similar to LPS or paclitaxel by dissociating LPS or paclitaxel from TLR4 in the model of white blood cell membrane chromatography (WB-CMC), and is a novel TLR4-antagonizing agent^18^,^19^,^20^.

In the study, we determined if AO-I can block paclitaxel-induced expression of pro-inflammatory cytokines and anti-apoptotic protein survivin, and potentiate paclitaxel–induced apoptosis and growth inhibition of EOC cells. We also performed a preliminary docking of AO-1 and paclitaxel to human MD-2 by computational simulation. We demonstrated, for the first time, that AO-I is able to sensitize EOC cells to paclitaxel by blocking MD-2-mediated TLR4/MyD88-dependent signaling pathway. The combination of AO-1 with paclitaxel elicites significantly greater inhibition of cell growth and more apoptosis, compared with paclitaxel alone.

## Results

### Impact of AO-1 on protein expression of MD-2, TLR4 and MyD88

**Figure 1:**
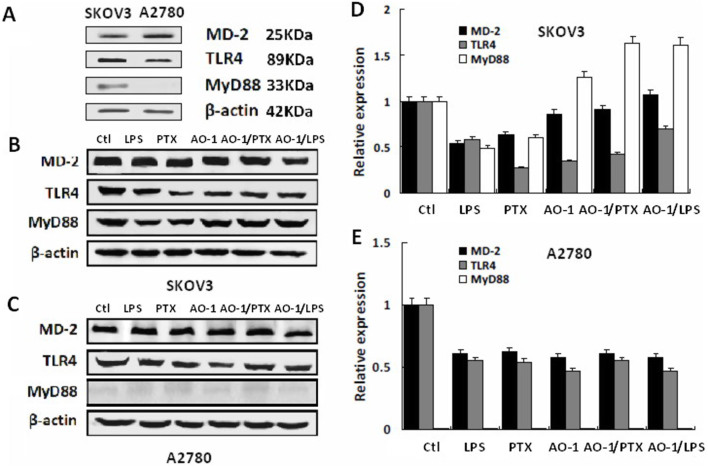
Expression of TLR4, MD-2 and MyD88 proteins by EOC cells treated with LPS, PTX and AO-1. (A). Expression of TLR4, MD-2 and MyD88 proteins by SKOV3 and A2780 cells (n = 3); (B–E). Protein expression of TLR4, MD-2 and MyD88 induced by LPS (1 μg/ml), paclitaxel (0.01 μmol/L) and AO-1(100 μmol/L), and by combination of LPS (1 μg/ml), paclitaxel (0.01 μmol/L) with AO-1 (100 μmol/L) in SKOV3 cells and A2780 cell, respectively (n = 3). Ctl and PTX represent the control and pacliaxel, respectively.

Western blot showed that TLR4 and MD-2 were expressed in both SKOV3 and A2780 cells, while MyD88 was expressed only in SKOV3 cells (Figure 1A). At 24 h post-LPS (1 μg/ml) and paclitaxel (0.01 μmol/L) treatment, protein levels of MD-2, TLR4 and MyD88 were significantly decreased (*P* < 0.05) in both SKOV3 and A2780 cells; AO-1 (100 μmol/L) decreased protein levels of MD-2 and TLR4 in both SKOV3 and A2780 cells, but significantly increased MyD88 protein level in SKOV3 cells (*P* < 0.05). Combination of AO-1 (100 μmol/L) with LPS (1 μg/ml) or paclitaxel (0.01 μmol/L) partially counteracted the LPS- or paclitaxel-induced decreased protein levels of MD-2 and TLR4, but significantly increased MyD88 protein level in SKOV3 cells but not in A2780 cells (*P* < 0.05) (Figure 1B–E). Signaling downstream of TLR4 in response to its ligands has been found to be different in the two cell lines^3^, suggesting that MyD88 plays a crucial role for receptor complex TLR4/MD-2 signaling in human EOC cells. Two EOC cell lines A2780 (MyD88^−^) and SKOV3 (MyD88^+^) were chosen for subsequent studies.

### AO-I downregulates paclitaxel-induced expression of VEGF and survivin

**Figure 2:**
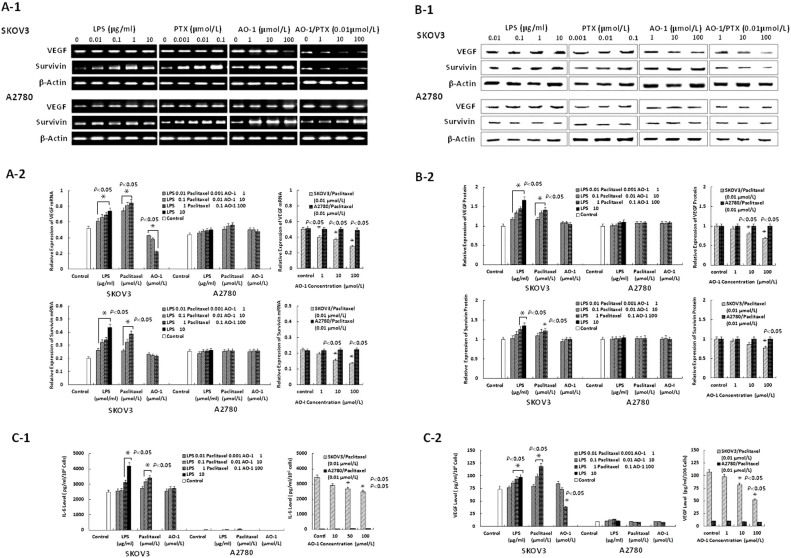
Expression of VEGF and survivin and levels of soluble IL-6 and VEGF. (A1–A2). The mRNA expression of VEGF and survivin induced by LPS, paclitaxel and AO-1 in SKOV3 cells and A2780 cells, respectively (n = 3); (B1–B2). The protein expression of VEGF and survivin induced by LPS, paclitaxel and AO-1 in SKOV3 cells and A2780 cells, respectively (n = 3); (C1–C2). Levels of soluble IL-6 and VEGF, induced by combination of paclitaxel with AO-1 in SKOV3 cells and A2780 cells, respectively (n = 3). * *P* < 0.05, compared to Control.

As shown in Figure 2A-1 and A-2 and in Figure 2B-1 and B-2, RT-PCR and Western blots analysis showed that SKOV-3 and A2780 cells constitutively expressed the mRNA and and protein of VEGF and survivin, and that LPS and paclitaxel significantly (*P* < 0.05) increased the expression of VEGF and survivin by SKOV-3 cells in concentration-dependent manner but not by A2780 cells; Treatment with AO-1 alone significantly decreased the constitutive mRNA expression of VEGF but not survivin by SKOV3 cells (Figure 2A-1 and A-2), and also markedly (*P* < 0.05) counteracted the paclitaxel-induced increased mRNA and protein expression of VEGF and survivin by SKOV-3 cells but not by A2780 cells in a concentration-dependent manner. These results suggest that downregulation of paclitaxel-induced expression of VEGF and survivin by AO-I requires MyD88-dependent TLR4 signaling in EOC cells.

### AO-I attenuates LPS- and paclitaxel-induced levels of soluble IL-6 and VEGF

The supernatants of both cell lines exposed to LPS or paclitaxel with or without addition of AO-I for 48 h were analyzed for levels of soluble IL-6 and VEGF by ELISA assay. As shown in Figure 2C-1 and C-2, LPS- or paclitaxel-treated SKOV3 cells secreted a wide range of IL-6 and VEGF (*P* < 0.05), but no or lower signal for soluble IL-6 and VEGF were detectable in A2780 cells; Interestingly, SKOV3 cells treated with AO-1 alone significantly decreased the level of VEGF (*P* < 0.05). Following treatment with AO-I, a significant decrease in paclitaxel-induced levels of soluble IL-6 and VEGF was observed in SKOV3 cells but not in A2780 cells (*P* < 0.05) (Figure 2C).

### AO-I enhances paclitaxel–induced growth inhibition

**Figure 3:**
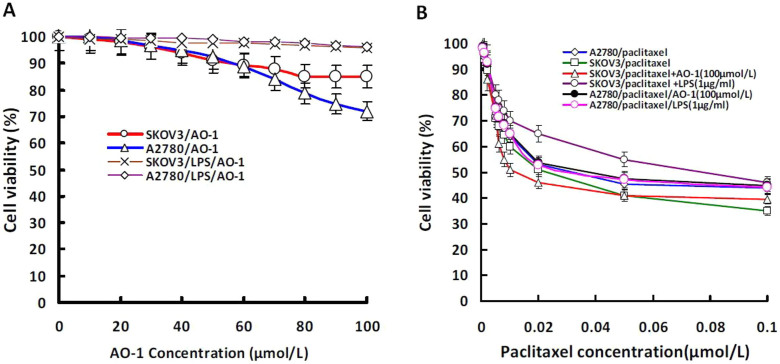
Proliferation of tumor cells in response to AO-I, paclitaxel or LPS. (A). Cell viabilty (%) of SKOV3 and A2780 cells treated with LPS (0.1 emsp14;μg/ml) alone or combined with AO-1 for 72 hours (n = 3); (B). Cell viabilty (%) of SKOV3 and A2780 cells treated with paclitaxel alone or combined with AO-1(100 μmol/L) or with LPS (0.1 μg/ml) (n = 3).

As shown in Table 1 and Figure 3A and 3B, SKOV3 cells showed more resistance to paclitaxel than A2780 cells (*P* < 0.05). AO-I or LPS and their combination did not markedly impact the proliferation of both SKOV3 and A2780 cells, the IC_50_ values of AO-I for SKOV3 and A2780 cells were far more than 100 μmol/L, suggesting that AO-I have no distinct cytotoxic activity against SKOV3 and A2780 cells when used alone. We also found that AO-I significantly enhanced paclitaxel-induced growth inhibition in SKOV3 cells, and in particular, decreased paclitaxel IC_50_ value by up to 3.5-fold (from 0.038 to 0.011 μmol/L) (*P* < 0.05), but had no sensitizing effect on A2780 cells when A2780 cells were treated with paclitaxel. In addition, LPS (0.1 μg/ml) greatly increased the resistance of SKOV3 cells to paclitaxel (*P* < 0.05). These results suggest that AO-I, as a potent antagonist of TLR4/MD-2 complex, could significantly potentiate the growth inhibitory effect of paclitaxel in MyD88^+^ EOC cells.

### AO-I potentiates paclitaxel-induced early apoptosis

To determine whether the decrease in cell viability is due to the increase of paclitaxel-induced early apoptosis, we measured Annexin V/PI binding responses of EOC cells for 24 hours. As shown in Table 2, paclitaxel induced the early apoptosis of both A2780 and SKOV3 cells. Following co-treatment with paclitaxel and AO-I, an approximate 2.5-fold increase in paclitaxel-induced apoptosis (Annexin V^+^ from 5.4% to 13.6%) was observed in SKOV3 cells, but the level of paclitaxel-induced early apoptosis of A2780 cells remained unchanged, suggesting that AO-I inhibits pro-survival response of paclitaxel that is MyD88-dependent in SKOV3 cells.

### Docking of AO-1 and paclitaxel to human MD-2

**Figure 4:**
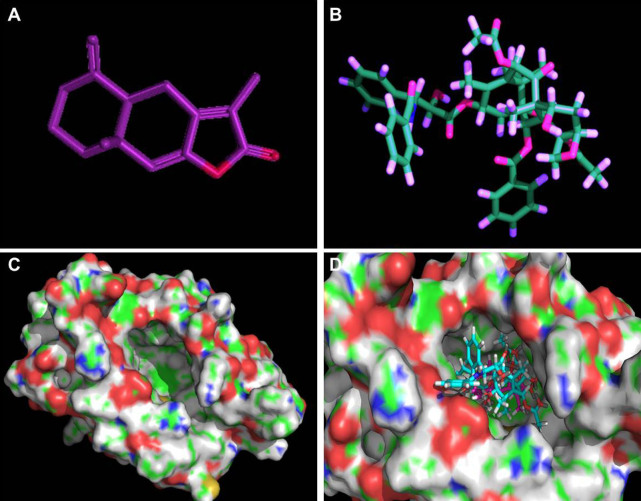
Molecular model of docking of paclitaxel and AO-I to the molecular model of MD-2. (A and B). 3D structures of AO-I and paclitaxel were drawn as a ball-and-stick representation: AO-I in purple, paclitaxel in blue-green. (C). 3D structure of human MD-2. Protein surface showing hydrophobic and hydrophilic properties. Green and red represent hydrophobicity and hydrophilicity, respectively, and the surface center of MD-2 with a potential binding-pocket (iron-gray); (D). AO-I binding to the hydrophobic pocket of MD-2, which partially overlaps with the binding site of paclitaxel.

We docked AO-1 and paclitaxel to the crystal structure of human MD-2 by computational simulation. AO-1 could be fitted into the hydrophobic pocket of human MD-2 and overlap with the binding site of paclitaxel (Figure 4), and competitively displace paclitaxel from MD-2 under the most energetically favorable simulation, suggesting that AO-1, as an antagonist, may be responsible for inhibition of TLR4-mediated paclitaxel signaling by binding to human MD-2.

## Discussion

The expression of TLR4/MyD88 signaling pathway linked to tumor progression and resistance to paclitaxel has been reported recently in EOC cells^3^,^7^,^11^. The expression of MyD88 in more than 70% of patients with EOC and has been identified as an indicator of tumor metastasis, paclitaxel chemoresistance and a significantly poor prognosis factor^9^,^11^. In respect to functioning TLR4/MyD88 signaling, evidence supporting its involvement in carcinogenesis and sensitivity to apoptosis mediated by paclitaxel is available for ovarian cancer^3^,^23^,^24^,^25^. Evidence implicates paclitaxel and LPS share a TLR4/MyD88-dependent pathway leading to activation of MAPK and NF-κB in generating pro-inflammatory cytokines^12^,^26^,^27^ and anti-apoptostic proteins in EOC cells. High level expression of IL-6 and VEGF and survivin are characterized by increasing survival and proliferation in EOC cells that express TLR4/MyD88, suggesting that activation of this pathway in EOC cells maintain chronic inflammation and promote cancer growth, metastasis and paclitaxel chemoresistance.

Ligand-induced MD-2-mediated dimerization of TLR4 is required for the activation of TLR4/MyD88-dependent signaling pathways. The MyD88-dependent response occurs on the dimerization of TLR4 which leads to the recruitment of MyD88 to the intracellular domain of TLR4, initiating the intracellular signal cascade that culminates in activation and nuclear translocation of transcription factors AP-1 and NF-κB leading to the induction of the expression of various inflammatory gene products^28^. MD-2 is a part of the TLR4 signaling complex with an indispensable role in activation of the TLR4 pathway and thus, the formation of TLR4/MD-2 complex may be one of the first lines of regulation in activating TLR4-mediated responses. It has been confirmed that LPS or paclitaxel binding to TLR4 results in dimerization of the TLR4 associated with MD-2^14^,^29^. MD-2 silencing has been reported to decrease LPS-induced cytokine production and TLR4/MyD88 pathway activity^30^. MD-2 undergoes a ligand-dependent conformational change that in turn induces or blocks the homotypic aggregation of TLR4/MD-2, followed by the recruitment of MyD88. The demonstration that paclitaxel can bind to TLR4/MD-2^31^ and therefore activate NF-κB could explain why tumor growth was observed during paclitaxel treatment of patient with advanced EOC^4^. Recent data attest to a role of MD-2 activity in colon cancer epithelial cell proliferation and migration, which may be important in the general correlation between innate immune response, chronic inflammation, and cancer^31^. Recent studies revealed that several small molecular including amitriptyline, curcumin and Eritoran, a structural analogue of LPS, exhibited TLR4 inhibition, possibly by binding pocket on interactions with binding pocket on MD-2, in a TLR4/MyD88-dependent manner^32^,^33^,^34^,^35^.

In this study, the expression of MD-2 and TLR4 were observed in both SKOV3 and A2780 cells but MyD88 only in SKOV3 cells. Indeed, our results showed that in SKOV3 cells, LPS and paclitaxel decreased protein levels of MD-1, TLR4 and MyD88. It is possible that the endocytosis of receptor complex TLR4/MD-2 following ligand-receptor interaction results in lysosomal degradation and E3-mediated degradation of Syk-phosphorylated MyD88^36^,^37^, leading to the control of intensity and duration of TLR4 signaling. Interestingly, in addition to reducing protein levels of MD-2 and TLR4, AO-1 also significantly increased protein levels of MyD88, suggesting that the inhibition of AO-1 on the TLR4/MyD88 signaling by blocking the binding of MD-2 to TLR4 may lead to the reduction of TGF-β1-mediated down-regulation of MyD88 expression^37^.

In response to paclitaxel, AO-1 can attenuate paclitaxel-induced increased expression of IL-6, VEGF and survivin, potentiate paclitaxel-induced growth inhibition and early apoptosis in MyD88^+^ EOC cells, suggesting that the binding of paclitaxel to MD-2 mediates dimerization of TLR4/MD-2 to activate MyD88-dependent signaling. But we found no significant cytotoxic effect in MyD88^+^ EOC cells treated with AO-I alone at less than 100 μmol/L, suggesting that AO-I, as an antagonist of TLR4/MyD88 signaling with low cytotoxicity, can sensitize MyD88^+^ EOC cells to paclitaxel. However, the underlying mechanisms of AO-I blocking TLR4 signaling are poorly understood. A triggering event on TLR4 is involved in the molecular rearrangement of the receptor complex and its homodimerization^14^,^38^,^39^. The assembly of the TLR4/MD-2 complex initiates a MyD88-dependent signaling cascade, which relocates NF-κB from the cytoplasm to the nucleus^40^.

Our preliminary docking analysis of AO-I and paclitaxel binding to the crystal structure of human MD-2 showed that AO-1 could preferentially fit into the hydrophobic binding pocket of human MD-2, which binds paclitaxel, suggesting that AO-1 induce the conformational changes of MD-2 that may obstruct the formation of an active TLR4/MD-2 complex, and then inactivate TLR4 signaling. Inhibition of MD-2-mediated active dimerization of TLR4 may be a promising therapic strategy for overcoming TLR4/MyD88 signaling-mediated resistance of EOC cells to paclitaxel. We demonstrated, for the first time, that the antagonizing effect of AO-I is TLR4/MD-2-mediated MyD88-dependent signaling pathway in EOC cells.

Our preliminary docking analysis of AO-I and paclitaxel binding to the crystal structure of human MD-2 showed that AO-1 could preferentially fit into to the hydrophobic binding pocket of human MD-2, suggesting that AO-1 induce the conformational changes of MD-2 that may obstruct the formation of an active TLR4/MD-2 complex, and then inactivate TLR4 signaling. We determined that AO-I binds to MD-2 at submicromolar affinity and competes functional cellular TLR4 signaling pathway stimulated by paclitaxel, and we demonstrated, by using FCM assay, that AO-1 can prevent paclitaxel- or LPS-induced formation of TLR4/MD-2 complex and cellular stimulation by interfering with TLR4/MD-2 binding (data not shown). Therefore, Inhibition of TLR4/MD-2 dimerization may be a promising therapic strategy for overcoming TLR4/MyD88 signaling-mediated resistance of EOC cells to paclitaxel. We demonstrated for the first time that the antagonizing effect of AO-I is TLR4/MD-2-mediated MyD88-dependent signaling pathway in EOC cells.

Taken together, these data indicate that the AO-1 inhibits TLR4/MyD88 signaling mediated pro-survival of paclitaxel in EOC cells and the combined use of AO-I with paclitaxel could improve tumor response to paclitaxel chemotherapy in patients with EOC by blocking MD-2-mediated activation of TLR4/MyD88 signaling.

## Methods

### Compounds and reagents

LPS, paclitaxel and MTT (3-[4,5-dimethylthiazol-2-yl]-2,5-diphenyl tetrazolium bromide) were purchased from Sigma Chemical Co. (St. Louis, MO, USA); AO-I, (4aS,8aS)-3,8a-dimethyl-5-methylidene-4a,6,7,8-tetrahydro-4H-benzo[f][1]benzofuran-2-one (CAS Number 73069-13-3, MF C15H18O2, MW (g/mol) 230.3022, HPLC ≥ 98%) was purchased from Chengdu Best-Reagent Co. Ltd. (Chengdu, Sichuan, China); the rabbit polyclonal antibodies to TLR4, MD-2 and MyD88 were purchased from Epitomics, Inc. (Burlingame, CA, USA) and Abcam plc. (Cambridge, MA, USA); the rabbit polyclonal antibodies to IL-6, VEGF, survivin and horseradish peroxidase conjugated secondary antibodies were purchased from Santa Cruz Biotechnology, Inc. (Santa Cruz, CA, USA).

### Cell lines and culture

Human EOC cell lines SKOV3 (TLR4+/MyD88+, derived from the ascites of a patient with advanced, metastatic EOC and resistant to most cytotoxic drugs)^41^ and A2780 (TLR4+/MyD88-, derived from a primary untreated and paclitaxel-sensitive cancer)^42^ were purchased from the Committee on Type Culture Collection of Chinese Academy of Sciences (CTCCCAS, Shanghai, China). Cell lines were maintained in RPMI 1640 medium (GIBCO) supplemented 10% heat-inactivated fetal calf serum (FCS), 2 mM L-glutamine, 100 U/ml penicillin and 40 IU/ml gentamicin at 37°C in a humidified atmosphere of 5% CO2 and 95% air. Subconfluent cells (80%) were passaged with a solution containing 0.25% trypsin and 0.5 mmol/L EDTA. Cell lines were tested for Mycoplasma and confirmed to be negative.

### Docking of AO-I or paclitaxel to the MD-2 structural model

Docking simulation of AO-I (CID: 5321018) and paclitaxel (CID: 36314) were carried out with the program AutoDock4 (open source, Scripps Research Institute), and the crystal structure of human MD-2 was cited from Protein Data Bank (2E56)^14^,^18^. The ligand-binding groove on MD-2 was kept rigid, whereas all torsible bonds of AO-I or paclitaxel were set free to perform flexible docking to produce more than 100 structures. Final docked conformations were clustered within the tolerance of 1 Å root-mean-square deviation.

### RNA isolation and RT-PCR

Total RNA was isolated from cell culture (1 × 10^6^ cells) using Trizol reagent (Invitrogen Co., CA, USA) according to the manufacturer's protocol. 2 μg of total RNA was reverse transcribed to cDNA. cDNA then amplified using the following primers specific for VEGF: F:5′-CACATAGGAGAGATGAGCTTC-3′, R: 5′-CCTCGGCTTGTCACATCTG-3′; for survivin: F: 5′-TCAAGGACCACCGCATC-3′, R: 5′-CAATCCATGGCAGCCAG-3′; for β-actin: F: 5′-AAGAGATGGCCACGGCTGCT-3′, R: 5′-GACTCGTCATACTCCTGCTTGCT-3′. PCR reaction was performed using the following conditions: Pre-denaturation at 95°C for 5 min. 35 cycles of amplification at 95°C for 15 sec. 56°C for 45 sec. and 72°C for 45 sec, and final extension at 72°C for 5 min. β-actinwas used as an internal control.

### Western blot analysis for protein expression

EOC cells were treated simultaneously with LPS or paclitaxel with or without addition of AO-I for different periods of time and lysed in RIPA buffer [1% Triton X-100, 150 mmol/L NaCl, 1 mmol/L EGTA, 50 mmol/L Tris–HCl, 0.1% sodium dodecyl sulfate (SDS), 1% sodium desoxycholate and phenylmethylsuphonyl fluoride (PMSF)]. Proteins separated by SDS-PAGE were electrotransfered to polyvinylidene difluoride (PVDF) membranes. The following Abs: anti-TLR4, anti-MD-2 and anti-MyD88 and anti-survivin were used for detection, and horseradish peroxidaseconjugated secondary antibodies (1:100,000 dilution) for development of reactions in a chemiluminescent detection system (ChemiDoc XRS+, Bio-Rad). β-actin antibodies were used as controls for equal protein loading.

### ELISA assay for cytokines

VEGF and IL-6 production was determined using Enzyme-linked immunosorbent assay (ELISA). Cells were plated in 12-well plates at 1 × 10^5^ cells/well in 1 mL of medium and treated simultaneously with LPS or paclitaxel with or without addition of AO-I for 24 h. The supernatants were collected, then the levels of IL-6 and VEGF were measured using ELISA kits (R&D Systems). The assay sensitivity varied from 5 to 15 pg/ml.

### Annexin V/PI binding for early apoptosis

A flow-based Annexin V/Propidium iodide (ANX-V/PI) assay was used to measure EOC cell early apoptosis. Briefly, cells were treated simultaneously with paclitaxel (0.01 μmol/L) with or without addition of AO-I (100 μmol/L) for 24 h, trypsinized with 0.25% trypsin, washed in PBS, resuspended in ANX V/PI-binding buffer and stained with 1 μg/mL FITC-conjugated ANX V and 1 μg/mL PI (keyGentec BioTHEC) for 15 min on ice in the dark. Cell apoptosis was evaluated by flow cytometry (BD FACS Canto II).

### Cell viability assay

To a 96-well plate, 5 × 10^3^ cells/well were pre-cultured for 24 hours, and then treated simultaneously with paclitaxel or LPS (1 μg/ml) with or without addition of AO-I, and 0.1% ethaol was used as a vehicle in triplicate. After 72 hours, 20 μl of MTT (5 mg/ml in PBS) was added to each well. The plates were gently shaken and incubated for 4 hours at 37°C in 5% CO_2_ atmosphere. The supernatant was removed and 200 μl of dimethyl sulfoxide (DMSO) was added and the plates were gently shaken to solubilize the formed formazan. Cell viability was determined by absorbance readings with ELISA Microplate Reader.

### Statistical analysis

Data was summarized using descriptive statistics of mean and standard deviation. ANOVA was used as a statistical test with SPSS 17.0 software, and *P* value < 0.05 was considered significant.

## Author Contributions

J.M.H.: Study design, manuscript editing; G.N.Z.: Study concepts, manuscript review; Y.S. and X.Z.: Quality control of data and algorithms; Y.Z.: Data analysis and interpretation, manuscript preparation; M.M.W., Q.L., W.W. and H.Y.L.: statistical analysis; S.Q.M., J.C. and B.F.D.: Data acquisition. All authors reviewed the manuscript.

## Figures and Tables

**Table 1:** Drug-induced growth inhibition in EOC cells in 4 treatment groups

		SKOV3 cells		A2780 cells	
Treatment group	*n*	IC_50_ (μmol/L)	P*	IC_50_ (μmol/L)	P*
AO-1	3	>100	-	>100	-
Paclitaxel	3	0.038 ± 0.004	-	0.024 ± 0.004	-
Paclitaxel/AO-I	3	0.011 ± 0.004*	0.027	0.029 ± 0.006	0.500
Paclitaxel/LPS	3	0.082 ± 0.002*	0.044	0.033 ± 0.004	0.100

EOC cells were treated with vehicle or paclitaxel plus AO-I (100 μmol/L) or paclitaxel plus LPS (1 μg/ml) for 72 hours. Relative number of viable cells was assayed using MTT. IC_50_ values were measured as a curvilinear regression equation for each survival curve. Data represent mean values ± S.D. from three independent experiments.

*P values, compared with paclitaxel alone.

**Table 2:** Drug-induced early apoptosis in EOC cells in 6 treatment groups

	SKOV3 cells		A2780 cells	
Treatment group	*%* AnnexinV^+^ cells	P*	*%* AnnexinV^+^ cells	P*
Control	4.0 ± 0.57	-	3.5 ± 0.40	-
AO-1(1 μmol/L)	4.3 ± 0.48	-	3.7 ± 0.38	-
AO-1(100 μmol/L)	5.1 ± 0.34	-	3.2 ± 0.44	-
Paclitaxe l (0.01 μmol/L)	5.8 ± 0.45	-	8.5 ± 0.65	-
Paclitaxel/AO-I (1 μmol/L)	9.9 ± 0.82*	0.002	9.1 ± 0.51	0.336
Paclitaxel/AO-I (100 μmol/L)	13.6 ± 1.76*	0.001	9.8 ± 1.01	0.593

EOC cells were treated with paclitaxel (0.01 μmol/L), AO-I (1 μmol/L, 100 μmol/L), and with paclitaxel (0.01 μmol/L) plus AO-I (1 μmol/L, 100 μmol/L) for 24 hours. FCM assay for AnnexinV binding as described in Materials and methods. Data represent mean values ± S.D. from three independent experiments.

*P values, compared with paclitaxel group, AO-1 groups and control group. AO-I significantly potentiates paclitaxel-induced early apoptosis of SKOV-3 cells in a concentration-dependent manner.
